# Effect of Finite-Size Heat Source’s Heat Capacity on the Efficiency of Heat Engine

**DOI:** 10.3390/e22091002

**Published:** 2020-09-08

**Authors:** Yu-Han Ma

**Affiliations:** 1 Graduate School of China Academy of Engineering Physics, No. 10 Xibeiwang East Road, Haidian District, Beijing 100193, China; yhma@gscaep.ac.cn; 2 Beijing Computational Science Research Center, Beijing 100193, China

**Keywords:** finite-size heat source, efficiency at maximum work, efficiency at maximum power

## Abstract

Heat engines used to output useful work have important practical significance, which, in general, operate between heat baths of infinite size and constant temperature. In this paper, we study the efficiency of a heat engine operating between two finite-size heat sources with initial temperature difference. The total output work of such heat engine is limited due to the finite heat capacity of the sources. We firstly investigate the effects of different heat capacity characteristics of the sources on the heat engine’s efficiency at maximum work (EMW) in the quasi-static limit. Moreover, it is found that the efficiency of the engine operating in finite-time with maximum power of each cycle is achieved follows a simple universality as $\eta =\eta_{C}/4+O\left(\eta_{C}^{2}\right)$, where $\eta_{C}$ is the Carnot efficiency determined by the initial temperature of the sources. Remarkably, when the heat capacity of the heat source is negative, such as the black holes, we show that the heat engine efficiency during the operation can surpass the Carnot efficiency determined by the initial temperature of the heat sources. It is further argued that the heat engine between two black holes with vanishing initial temperature difference can be driven by the energy fluctuation. The corresponding EMW is proved to be $\eta_{\mathrm{MW}}=2-\sqrt{2}$.

## 1. Introduction

As one of the most useful devices in modern society, the heat engine converts the heat extracted from the heat sources into useful work, which is one of the core fields in thermodynamic research [1,2,3,4,5,6,7,8,9]. Early heat engine research was limited to reversible cycles in the quasi-static limit, with which, as stated by the Carnot’s theorem [1], the achievable maximum efficiency of heat engines is the so-called Carnot efficiency $\eta_{C}=1-T_{L}/T_{H}$, where $T_{H}$ ($T_{L}$) is the temperature of the hot (cold) bath. Since the last century, with the maturity of quantum theory and its related technologies, people started to pay attention to the performance of quantum heat engines working in micro-scale within the framework of quantum thermodynamics [4,5,8,9,10,11,12,13,14,15,16,17,18,19]. Series of the quantum effect, such as coherence, entanglement, quantum phase transition, etc., of the working substance or heat source have been studied to realize better heat engines [13,20,21,22,23,24,25,26,27]. On the other hand, with the development of non-equilibrium thermodynamics [2,3,28], the optimization of actual heat engines under the framework of finite-time thermodynamics attracted a wide range of attention [29,30,31,32,33]. Extensive research on the efficiency at maximum power (EMP) [34,35,36,37,38,39,40,41,42], trade-off relation between power and efficiency [43,44,45,46], and optimal operation of heat engine have been proposed [47,48]. The motivation for these studies stems from the fact that time is a finite resource, we cannot trade at the cost of infinitely long working time for heat engines that are efficient but have vanishing output power.

Most of studies about heat engines have regarded the heat sources as an infinite system, which means, the sources can continuously provide or absorb heat. However, just like time, the realistic heat source is also a finite resource, so it is an interesting and practical task to consider the optimization of the heat engine working between finite-size heat sources. Recently, people began to consider this issue with different perspectives. For example, considering the Carnot heat engine working between finite heat sources [49,50,51], linear irreversible heat engines working in finite time with finite-size bath [52,53], and the bounds of optimal efficiency the engines can achieve [54,55]. In addition, the influences of the finite-size heat source on the quantum heat engine [56,57,58,59,60] and quantum battery [61] also attract some attention. In general, the finite-size effect of the heat source is reflected in the limited heat capacity. Therefore, the feature of the heat capacity of the heat sources will directly determine the performance of the heat engine working between them. In this paper, we discuss the effects of finite-size heat sources with different heat capacity characteristics on heat engine’s efficiency, in both quasi-static and finite-time circumstances. Several bounds for the efficiency in different situations with finite operation time or finite-size sources are obtained as illustrated in Table 1. In particular, we study the case where the heat source has a negative heat capacity, which is proved to be advantageous for improving the efficiency of the heat engine. To our best knowledge, this has never been reported before.

The paper is organized as follows: In Section 2, we first generally discuss the influence of the heat capacity of the finite-size heat sources on the efficiency of the heat engine at maximum work (EMW) with quasi-static cycle. Then the efficiency of the heat engine in the high and low temperature regime with different heat capacity function are derived. In the low-temperature regime, it is found that the dimension of the heat source will influence the EMW of the heat engine, and the higher EMW can be achieved with higher dimension materials. In Section 3, the study in quasi-static situation is extend to the finite-time case, where we consider the low-dissipation Carnot-like heat engine working between two finite-size sources. We point out that when the heat engines operates with maximum power in each cycle, the efficiency of the whole process follows a simple universality as $\eta =\eta_{C}/4+O\left(\eta_{C}^{2}\right)$, where $\eta_{C}$ is the Carnot efficiency determined by the initial temperature of the sources. With the black hole as an illustration, we study the negative heat capacity system service as the finite-size heat source in Section 4. Conclusions and discussions are given in Section 5.

## 2. Heat Engine Working between Finite-Size Heat Source

**Figure 1:**
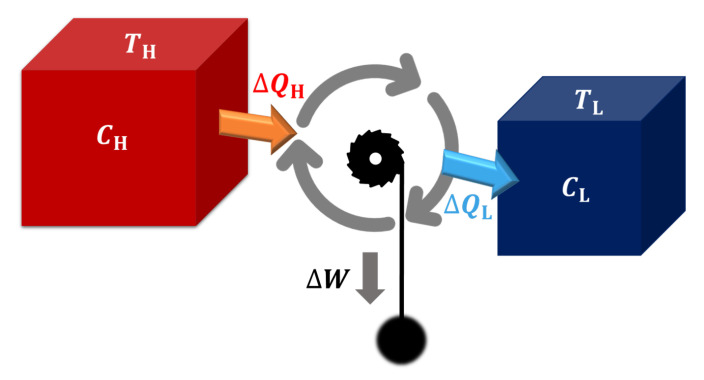
Heat engine working between two finite-size heat sources. $T_{H}$ ($T_{L}$) and $C_{H}$ ($C_{L}$) are the temperature and heat capacity of the high (low) temperature source H (L), respectively. $W=Q_{H}-Q_{L}$ is the output work of the engine per cycle while $Q_{H}$ is the heat absorbed from the hot source and $Q_{L}$ the heat releases to the cold source.

As shown in Figure 1, we consider the heat engine of interest is working between two heat source H and L with finite size, where $T_{H}$ ($T_{L}$) and $C_{H}$ ($C_{L}$) are the temperature and heat capacity of the high (low) temperature source H (L), respectively. The initial temperature of the hot (cold) source is denoted as $T_{H}^{\left(0\right)}$ ($T_{C}^{\left(0\right)}$). When the working substance WS run in an arbitrary thermodynamic cycle, the heat engine can generate output work $W^{\left(i\right)}=\int_{t_{i}}^{t_{i+1}}\dot{W}dt$ in the *i*-th cycle, where $t_{i}$ is the start time of such cycle ($t_{1}=0$). Accoding to the first law of thermodynamics, $W^{\left(i\right)}=Q_{H}^{\left(i\right)}-Q_{L}^{\left(i\right)}$. Here $Q_{H}^{\left(i\right)}=\int_{t_{i}}^{t_{i+1}}{\dot{Q}}_{H}dt$ is the heat absorbed from the high temperature source, and $Q_{L}^{\left(i\right)}=\int_{t_{i}}^{t_{i+1}}{\dot{Q}}_{L}dt$ is the heat releases to the low temperature heat source. When the heat engine runs for *k* cycles, we can evaluate the heat engine’s performance with its efficiency η and power *P*, namely,

(1) $$\begin{array}{cc} \eta =\eta (t_{k+1}) & =\frac{\sum_{i=1}^{i=k}W^{\left(i\right)}}{\sum_{i=1}^{i=k}Q_{H}^{\left(i\right)}}=\frac{\int_{0}^{t}\dot{W}dt}{\int_{0}^{t}{\dot{Q}}_{H}dt}, \end{array}$$

(2) $$\begin{array}{cc} P=P(t_{k+1}) & =\frac{\sum_{i=1}^{i=k}W^{\left(i\right)}}{\sum_{i=1}^{i=k}\tau_{i}}=\frac{\int_{0}^{t}\dot{W}dt}{t} \end{array}$$

where $\tau_{i}=t_{i+1}-t_{i}$ is the cycle time, $t_{k+1}=\sum_{i=1}^{i=k}\tau_{i}$ is the engine’s total working time from the 1-th cycle to the *k*-th cycle, and we have replaced $\sum_{i=1}^{i=k}\int_{t_{i}}^{t_{i+1}}$ to $\int_{0}^{t}$ for convenience. For the size of these two heat sources approach infinity as well as the heat capacity, i.e., $C_{H,L}\to \infty$, their temperature will remain constant as the initial time, namely, $T_{H}=T_{H}^{\left(0\right)}$ and $T_{L}=T_{L}^{\left(0\right)}$. In such case, the two heat sources service as two heat bath, and the maximum efficiency of the heat engine is bounded by the well-known Carnot efficiency

(3) $$\eta_{C}=1-\frac{T_{L}^{\left(0\right)}}{T_{H}^{\left(0\right)}},$$

which can be achieved when the working substance works under a reversible Carnot cycle with vanishing output power. However, for the heat sources with finite size, their temperature will change as the heat engine works. Specifically, after providing heat to the working substance, $T_{H}$ of the high-temperature heat source decreases, while, on the other hand, the low-temperature source’s temperature $T_{L}$ increases since the working substance releases heat to it. Here we have assumed $C_{H,L}>0$ since most physical systems have positive heat capacity, while the extreme special situation where the heat sources have negative heat capacity, i.e., $C_{H,L}<0$ will be discussed in Section 4. As the time goes by, the heat engine will finally stop outputting work when the temperature of the two heat sources become the same, namely, $T_{H}\left(t_{f}\right)=T_{L}\left(t_{f}\right)$, where $t_{f}$ is introduced as the stop time of the heat engine. In this case, the output work of the engine has achieved its maximum, i.e., $W\left(t\right)\leq W\left(t_{f}\right)\equiv W_{\max}$, as the heat engine cannot continue operating after the heat source temperature reaches the same. The corresponding efficiency

(4) $$\eta \left(t_{f}\right)=\frac{\int_{0}^{t_{f}}\dot{W}dt}{\int_{0}^{t_{f}}\dot{Q_{H}}dt}=\frac{W_{\max}}{\int_{0}^{t_{f}}\dot{Q_{H}}dt}$$

is called the efficiency at maximum work (EMW) [52,62] and we denote it as $\eta \left(t_{f}\right)\equiv \eta_{\mathrm{MW}}$ thereafter. Here, we have assumed that the heat engine can run *N* complete cycles, thus $t_{f}=\sum_{i=1}^{i=N}\tau_{i}$. In the following discussion, we focus on the situation that the heat engine can work with limited but sufficient cycles, i.e., N≫1. With the endo-reversible condition, we have $\dot{Q_{L}}=T_{L}{\dot{S}}_{L}$, and $\dot{Q_{H}}=-T_{H}{\dot{S}}_{H}$, where $S_{L}$ and $S_{H}$ are respectively the entropy of the low-temperature and high-temperature sources. Such that

(5) $$\eta_{\mathrm{MW}}=1-\frac{\int_{0}^{t_{f}}\dot{Q_{L}}dt}{\int_{0}^{t_{f}}\dot{Q_{H}}dt}=1+\frac{\int_{0}^{t_{f}}T_{L}{\dot{S}}_{L}dt}{\int_{0}^{t_{f}}T_{H}\dot{S_{H}}dt}$$

With the operation of the heat engine, noticing the temperature difference of the two source become smaller and smaller (for finite positive heat capacity sources), and $T_{L}>T_{L}^{\left(0\right)}$, $T_{H}<T_{H}^{\left(0\right)}$, thus we have [52]

(6) $$\eta_{\mathrm{MW}}<1+\frac{T_{L}^{\left(0\right)}\int_{0}^{t_{f}}{\dot{S}}_{L}dt}{T_{H}^{\left(0\right)}\int_{0}^{t_{f}}\dot{S_{H}}dt}\leq \eta_{C},$$

where the equal sign on the right side only hold in the reversible limit with $\int_{0}^{t_{f}}{\dot{S}}_{L}dt=-\int_{0}^{t_{f}}\dot{S_{H}}dt$ [1,2]. The above discussion implies that, the finite heat capacity of the heat sources and the irreversibility of the cycle limit the EMW the heat engine can achieve. Therefore, the following two questions naturally raises: (i) What is the heat engine’s maximum EMW when working at two finite-size heat bath? (ii) How the specific feature of the heat source’s heat capacity affects such EMW? In the following of this section, we focus on the reversible case with quasi-static cycles, and the effect of irreversibility of the cycle in the finite-time case will be further studied in Section 3. We first rewrite the efficiency of Equation (5) in term of $C_{H}$ and $C_{L}$ as

(7) $$\eta_{\mathrm{MW}}=1+\frac{\int_{0}^{t_{f}}C_{L}{\dot{T}}_{L}dt}{\int_{0}^{t_{f}}C_{H}{\dot{T}}_{H}dt},$$

where $\dot{Q_{H}}=-C_{H}\dot{T_{H}}$ and $\dot{Q_{L}}=C_{L}\dot{T_{L}}$ have been used for the two heat sources. For a given physical system, heat capacity is generally the function of temperature, i.e., $C_{H,L}=C_{H,L}\left(T\right)$, and one can complete the integral in Equation (7) explicitly with the specific form of $C_{H,L}\left(T\right)$. Assuming the heat capacity of the sources follow the Debye’s Law [63], for example most of the crystal, such that $C\left(T\right)=$ const in the high temperature regime of T/Θ≫1, and $C\left(T\right)\propto T^{n}$ in the low-temperature regime of T/Θ≪1. Here Θ and *n* are the Debye temperature and the dimension of material, respectively. In the following discussion, we focus on these two regimes.

### 2.1. High Temperature Regime

In this case, the heat capacity only determined by the size (particle number) of the source, such that Equation (7) is simplified as

(8) $$\eta_{\mathrm{MW}}=1-\frac{C_{L}\left[T_{L}\left(t_{f}\right)-T_{L}^{\left(0\right)}\right]}{C_{H}\left[T_{H}^{\left(0\right)}-T_{H}\left(t_{f}\right)\right]},$$

where $T_{L}\left(t_{f}\right)=T_{H}\left(t_{f}\right)\equiv T_{E}$ is the equilibrium temperature of the two sources. The EMW is achieved with the reversible cycle, in which no irreversible entropy is generated, i.e., $\sum_{\theta =H,L,\mathrm{WS}}\int_{0}^{t_{f}}\dot{S_{\theta }}dt=0$. Here $S_{\mathrm{WS}}$ is the entropy of the working substance. Assuming the size of the heat sources are much lager than the working substance, namely $C_{\mathrm{WS}}/C_{\alpha }\ll 1$ (α=H,L), the entropy change of the working substance $S_{\mathrm{WS}}=\int_{0}^{t_{f}}\dot{S_{\mathrm{WS}}}dt$ can be ignored in comparision with that of the two sources, such that

(9) $$\int_{0}^{t_{f}}\left(\frac{\dot{Q_{H}}dt}{T_{H}}+\frac{\dot{Q_{C}}dt}{T_{C}}\right)=0,$$

where the endo-reversible condition is used for the two sources. The above equation can be further written as, in terms of the heat capacity,

(10) $$\int_{T_{L}^{\left(0\right)}}^{T_{E}}\frac{C_{L}dT_{L}}{T_{L}}+\int_{T_{H}^{\left(0\right)}}^{T_{E}}\frac{C_{H}dT_{H}}{T_{H}}=0.$$

which is the reversible condition of the whole process. Such condition is satisfied with $\tau_{i}\gg t_{r}$(i=1,2,…,N), where the ≫ guarantees each cycle meet the quasi-static condition and thus the engine works reversibly, $t_{r}$ is the characteristic time for the working substance relaxing to thermal equilibrium with the heat source, which is determined by the coupling strength between the working substance and the heat source. Finish the integral in Equation (10), we find the equilibrium temperature

(11) $$T_{E}=T_{H}^{\left(0\right)}{\left(1-\eta_{C}\right)}^{\frac{\xi }{\xi +1}},$$

where $\xi \equiv C_{L}/C_{H}$ is defined as the heat capacity ratio between the low and high temperature heat source, which characterizes the asymmetry of two heat sources. By substituting Equation (11) into Equation (8), the EMW is obtained in terms of ξ and $\eta_{C}$ as

(12) $$\eta_{\mathrm{MW}}=1-\frac{\xi \left[{\left(1-\eta_{C}\right)}^{\frac{\xi }{\xi +1}}-\left(1-\eta_{C}\right)\right]}{1-{\left(1-\eta_{C}\right)}^{\frac{\xi }{\xi +1}}}.$$

**Figure 2:**
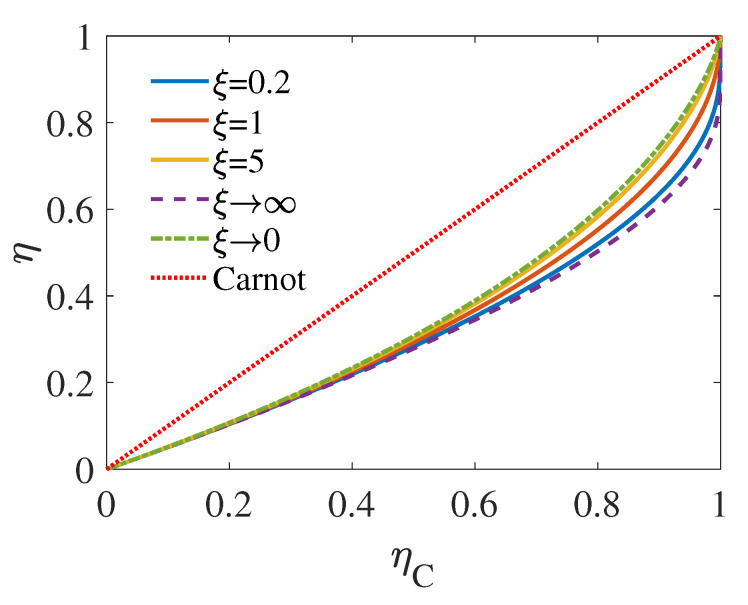
EMW $\eta_{\mathrm{MW}}$ as the function of $\eta_{C}$ with different $\xi =C_{L}/C_{H}.$ The curves relate to infinite heat capacity (in comparison with the hot one) of low-temperature source (ξ→∞) and infinite heat capacity (in comparison with the cold one) of high-temperature source (ξ→0) are given by Equation (13). The other are plot with Equation (12).

Here $\eta_{C}=1-T_{L}/T_{H}$ is the Carnot efficiency determined by the initial temperature of the sources. We illustrated $\eta_{\mathrm{MW}}$ as the function of $\eta_{C}$ in Figure 2. In three different limit case, namely, the symmetry case with ξ=1, infinite heat capacity (in comparison with the hot one) of the low-temperature source with ξ→∞, and infinite heat capacity (in comparison with the cold one) of the high-temperature source with ξ→0, we simplify Equation (12) as follows

(13) $$\eta_{\mathrm{MW}}=\left\{\begin{array}{cc} 1-\sqrt{1-\eta_{C}} & \xi =1\\ 1+\left(\eta_{C}^{-1}-1\right)\ln\left(1-\eta_{C}\right) & \xi \to \infty \\ 1+\eta_{C}\ln^{-1}\left(1-\eta_{C}\right) & \xi \to 0 \end{array}\right.$$

As shown in Figure 2, the maximum EMW is achieved with ξ→∞, which have also been reported in [49]. Although, infinity is never reached in practice, one can find in Figure 2 that, when ξ=5 (the yellow solid curve), the EMW already gets pretty close to the that of ξ→∞, as represented by the green dashed line. It can also be seen from Figure 2 that for a given $\eta_{C}$, $\eta_{\mathrm{MW}}$ is an increasing function of ξ, which indicates that, in the case where the hot source with smaller heat capacity than the cold one, the engine would operate with higher EMW.

Moreover, it can be proved from Equation (12) that the $\eta_{\mathrm{MW}}$ in different limits of ξ have the same coefficient in the first order of $\eta_{C}$, namely, $\eta_{\mathrm{MW}}=\eta_{C}/2+O\left(\eta_{C}^{2}\right)$, which shares the same universality with efficiency at maximum power [32,41,64]

### 2.2. Low Temperature Regime

Different from the above case within the high-temperature regime, the capacity of the heat sources in the low-temperature regime of T/Θ≪1, according to the Debye’s law, follows $C_{H,L}\left(T\right)=\Lambda_{H,L}T^{n}$. Here, $\Lambda_{H,L}$ is the heat source size-dependent coefficient, and *n* is the dimension of the sources. The reversible condition of Equation (10) in this case becomes

(14) $$\int_{T_{L}^{\left(0\right)}}^{T_{E}}\Lambda_{L}T_{L}^{n-1}dT_{L}+\int_{T_{H}^{\left(0\right)}}^{T_{E}}\Lambda_{H}T_{H}^{n-1}dT_{H}=0,$$

namely,

(15) $$\Lambda_{L}\left(T_{E}^{n}-T_{L}^{n}\right)=\Lambda_{H}\left(T_{H}^{n}-T_{E}^{n}\right),$$

which gives the equilibrium temperature

(16) $$T_{E}={\left(\frac{{\left(T_{H}^{\left(0\right)}\right)}^{n}+\xi {\left(T_{L}^{\left(0\right)}\right)}^{n}}{1+\xi }\right)}^{\frac{1}{n}}$$

where $\xi =C_{L}\left(T\right)/C_{H}\left(T\right)=\Lambda_{L}/\Lambda_{H}$ is the capacity ratio of the two sources at the same temperature. Combining Equations (7) and (16), the EMW at low temperature regime reads

(17) $$\eta_{\mathrm{MW}}=1-\frac{\xi \chi -\xi {\left(1-\eta_{C}\right)}^{n+1}}{1-\chi },$$

where

(18) $$\chi ={\left[\frac{1+\xi {\left(1-\eta_{C}\right)}^{n}}{1+\xi }\right]}^{\frac{n+1}{n}}$$

In the limit of ξ→∞, keeping the first order of $\xi^{-1}$ in χ, we obtain

(19) $$\eta_{\mathrm{MW}}\left(\xi \to \infty \right)=1-\frac{n+1}{n}\left[1-\frac{\eta_{C}}{1-{\left(1-\eta_{C}\right)}^{n+1}}\right].$$

On the other hand, in the case of ξ→0, we expand χ up to the first order of ξ and find that

(20) $$\eta_{\mathrm{MW}}\left(\xi \to 0\right)=1-\frac{n}{n+1}\left[\frac{1-{\left(1-\eta_{C}\right)}^{n+1}}{1-{\left(1-\eta_{C}\right)}^{n}}\right]$$

**Figure 3:**
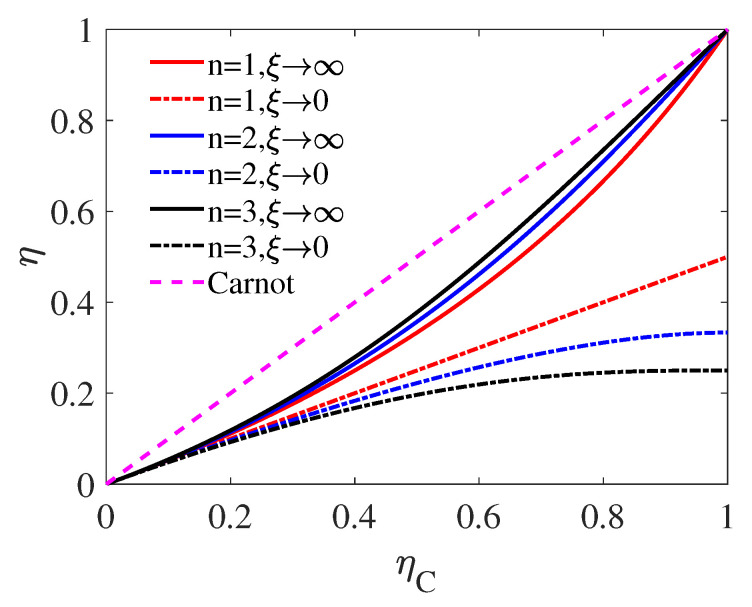
EMW $\eta_{\mathrm{MW}}$ as the function of $\eta_{C}$ with different source dimension *n*. The curves relate to ξ→∞ and ξ→0 are given by Equation (19) and Equation (20), respectively.

In Figure 3, the EMW in these two limit cases are plotted as the function of $\eta_{C}$ with the source dimension n=1,2,3. The curves shows that $\eta_{\mathrm{MW}}\left(\xi \to \infty \right)$, the upper bound of $\eta_{\mathrm{MW}}$, increases with the heat source dimension *n*; while the lower bound of $\eta_{\mathrm{MW}}$, i.e., $\eta_{\mathrm{MW}}\left(\xi \to 0\right)$ decreases with *n*. This means that, theoretically, we can use heat sources with higher dimension to achieve higher efficiency.

Particularly, when the considered heat source is one-dimensional, i.e., n=1, Equation (17) is directly simplified as

(21) $$\eta_{\mathrm{MW}}\left(n=1\right)=\frac{\eta_{C}}{2-\kappa \eta_{C}},$$

where $\kappa =\xi /\left(1+\xi \right),\kappa \in \left(0,\infty \right)$. Since $\eta_{\mathrm{EMW}}\left(n=1\right)$ is a monotonically increasing function of κ, we conclude that

(22) $$\eta_{L}\equiv \frac{\eta_{C}}{2}\leq \eta_{\mathrm{MW}}\left(n=1\right)\leq \frac{\eta_{C}}{2-\eta_{C}}\equiv \eta_{U}.$$

Similar result has been found by [55] with the energy-entropy relation. In addition, in the limit of $\eta_{C}\to 1$, the lower bound of $\eta_{\mathrm{MW}}$ in Equation (20) is found to be only determined by the heat source dimension, i.e.,

(23) $$\lim_{\eta_{C}\to 1}\eta_{\mathrm{MW}}\left(\xi \to 0\right)=\frac{1}{n+1}.$$

**Figure 4:**
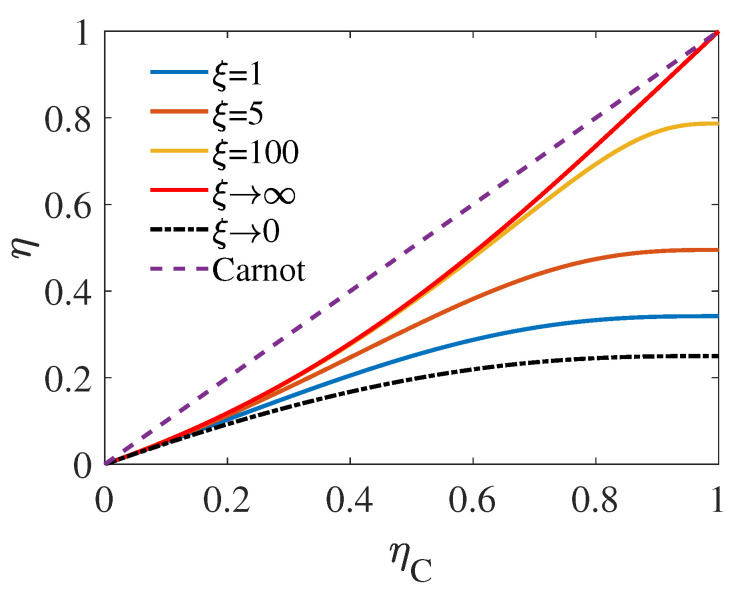
EMW $\eta_{\mathrm{MW}}$ as the function of $\eta_{C}$ with different ξ for source dimension n=3. The curves with finite ξ are plotted with Equation (17), while the curves relate to ξ→∞ and ξ→0 are given by Equation (19) and Equation (20), respectively.

This phenomenon is observed in Figure 3, where the intersection of the three dash-dotted lines and $\eta_{C}=1$ are, from top to bottom, $\eta_{\mathrm{MW}}=1/2$, $\eta_{\mathrm{MW}}=1/3$, and $\eta_{\mathrm{MW}}=1/4$, respectively. We also plot the EMW as the fucntion of $\eta_{C}$ with different ξ for source dimension fixed at n=3 in Figure 4. As demonstrated by Figure 4, similar to Figure 2 corresponding to the high-temperature regime, EMW in the low-temperature regime also increases with ξ. However, unlike the high-temperature behavior, when $\eta_{C}$ is relatively large, $\eta_{\mathrm{MW}}$ with ξ of very large value can approach the upper bound of EMW, i.e., $\eta_{\mathrm{MW}}\left(\xi \to \infty \right)$. It can be seen from the figure that when ξ=100, there is still a significant distance between the upper bound of EMW (red solid curve) and $\eta_{\mathrm{MW}}\left(\xi =100\right)$ (yellow solid curve) in the large-$\eta_{C}$ regime. Moreover, as the result of Equation (17), up to the first order of $\eta_{C}$, an universality is found for the EMW as

(24) $$\eta_{\mathrm{MW}}=\frac{\eta_{C}}{2}+O\left(\eta_{C}^{2}\right),$$

which is hold in both low-temperature regime (Equation (17)) and high-temperature regime (Equation (12)). Such an universality has been discovered before for EMP [32,41,64].

For those systems that do not satisfy the Debye’s law, we can first derive the heat capacity as the function of temperature from its energy spectrum and the corresponding equilibrium population. Then taking using of Equations (7) and (10) to obtain the EMW in quasi-static limit. It should be mentioned that the general result of Equation (17) is also suitable for Fermi gas at low temperature and photon(black body radiation) by taking n=1 and n=3, respectively. Since the capacity of the former follows $C_{\mathrm{FG}}\left(T\right)\propto T$, while $C_{\mathrm{PG}}\left(T\right)\propto T^{3}$ for the latter [65].

## 3. Finite-Time Performance of the Heat Engine

In the previous section, we derive the EMW of heat engines operating under the reversible condition, which is satisfied by the quasi-static cycles with almost vanishing output power. In this section, we extend our discussion in Section 2 to the finite-time case [52,53]. Note that, in such situation, the heat engine no longer operates in the reversible cycles, thus the irreversibility of the heat engine, characterized by the irreversible entropy generation [2], should be taken into consideration.

Unlike the optimization goal of Refs. [52,53], where the efficiency at maximum time-average power of the whole process is studied, we focus on how efficient the engine can be when the power of each cycle is maximized. Similar to the situation in Section 2, the engine of interest can run *N* cycle from t=0 to the stop time $t=t_{f}$, where N≫1 but finite as we assumed before. The operation time of the *i*-th cycle (i=1,2,…N) begins at $t=t_{i}$ is denoted as $\tau_{i}=t_{i+1}-t_{i}$. It follows from Equation (1) that the efficiency of the whole process then reads

(25) $$\eta =1-\frac{\sum_{i=1}^{N}Q_{L}^{\left(i\right)}}{\sum_{i=1}^{N}Q_{H}^{\left(i\right)}},$$

where $Q_{H}^{\left(i\right)}=\int_{t_{i}}^{t_{i+1}}{\dot{Q}}_{H}dt$ and $Q_{L}^{\left(i\right)}=\int_{t_{i}}^{t_{i+1}}{\dot{Q}}_{L}dt$. In the *i*-th cycle, the output power is $P^{\left(i\right)}=\left(Q_{H}^{\left(i\right)}-Q_{L}^{\left(i\right)}\right)\tau_{i}^{-1}$. Below we try to obtain the upper and lower bounds for η under the condition that the maximum $P^{\left(i\right)}$ is achieved in each cycle. Suppose the heat engine works in the finite-time Carnot-like cycle, which contains two adiabatic processes and two finite-time isothermal process. In the finite-time isothermal processes, the heat transfer generally follows, in the the *i*-th cycle,

(26) $$Q_{H}^{\left(i\right)}=T_{H}^{\left(i\right)}S_{\mathrm{re}}^{\left(i\right)}-Q_{\mathrm{irr},H}^{\left(i\right)},$$

(27) $$Q_{L}^{\left(i\right)}=T_{L}^{\left(i\right)}S_{\mathrm{re}}^{\left(i\right)}+Q_{\mathrm{irr},L}^{\left(i\right)}.$$

Here, $T_{H}^{\left(i\right)}$($T_{L}^{\left(i\right)}$) is the temperature of the high (low) temperature heat source, which can be considered as a constant in the whole process of the *i*-cycle, since the temperature of the heat source changes very little in each cycle with the assumption N≫1. $S_{\mathrm{re}}^{\left(i\right)}$ is reversible entropy change, and $Q_{\mathrm{irr},\alpha }^{\left(i\right)}$ (α=H,L) is the irreversible heat transfer relates to the corresponding source. With the low-dissipation assumption, as suggested first by Esposito et al. [41], the irreversible heat transfer follows the 1/τ scaling as $Q_{\mathrm{irr},\alpha }^{\left(i\right)}=\Gamma_{\alpha }^{\left(i\right)}/\tau_{\alpha }^{\left(i\right)}$, where $\Gamma_{\alpha }^{\left(i\right)}$ depends on the dissipative feature of the working substance when contacting with the heat source [46] and $\tau_{\alpha }^{\left(i\right)}$ is the operation time of the corresponding process. Such scaling has been proved for both classical [66] and quantum system [46,47] and was observed in some recent experiments [48,67]. Applying straightforward optimization of $P^{\left(i\right)}\left(\tau_{H}^{\left(i\right)},\tau_{L}^{\left(i\right)}\right)$ with respect to $\tau_{H}^{\left(i\right)}$ and $\tau_{L}^{\left(i\right)}$, the EMP in the *i*-th cycle is obtained as [41] (see Appendix A for detailed derivation)

(28) $$\eta_{\mathrm{MP}}^{\left(i\right)}=\frac{\eta_{C}^{\left(i\right)}}{2-\gamma^{\left(i\right)}\eta_{C}^{\left(i\right)}},$$

where

(29) $$\gamma^{\left(i\right)}={\left(1+\sqrt{\Gamma_{L}^{\left(i\right)}/\Gamma_{H}^{\left(i\right)}}\right)}^{-1}\in \left[0,1\right]$$

and $\eta_{C}^{\left(i\right)}=1-T_{L}^{\left(i\right)}/T_{H}^{\left(i\right)}$ is the Carnot efficiency determined by the temperature of the sources in the *i*-th cycle. It is worth mentioning that the optimal operation time related to the above EMP of each cycle relies on the corresponding temperature of the sources (for a detailed deviation, please see Equations (A5) and (A6) of Appendix A). When the maximum power output for each cycle is achieved, the efficiency of Equation (25), denoted as $\eta^{\mathrm{FT}}$, becomes

(30) $$\eta^{\mathrm{FT}}=\frac{\sum_{i=1}^{N}\eta_{\mathrm{MP}}^{\left(i\right)}Q_{H}^{\left(i\right)}}{\sum_{i=1}^{N}Q_{H}^{\left(i\right)}}.$$

For simplicity, we only consider the heat source with constant heat capacity in the following. As we studied In Section 2, the EMW in the reversible situation is bounded as $\eta_{\mathrm{MW}}\left(\xi \to 0\right)\leq \eta_{\mathrm{MW}}\leq \eta_{\mathrm{MW}}\left(\xi \to \infty \right)$ (see Equation (13)). Therefore, to achieve higher efficiency in finite time, we should focus on the case of ξ→∞, where the heat capacity of the low-temperature source is much lager than that of the high-temperature one, such that the cold source is kept at a constant temperature in the whole process, i.e., $T_{L}^{\left(i\right)}=T_{L}^{\left(0\right)}$ and the hot source temperature decreases with time until $T_{H}^{\left(N\right)}=T_{L}^{\left(0\right)}$ and the heat engine stops working. Substituting Equation (28) into Equation (30), and replace the sum by integral with N≫1, we obtain

(31) $$\eta^{\mathrm{FT}}=\frac{\int_{0}^{t_{f}}\frac{\eta_{C}\left(t\right)}{2-\gamma \eta_{C}\left(t\right)}{\dot{Q}}_{H}dt}{\int_{0}^{t_{f}}{\dot{Q}}_{H}dt},$$

where we have assumed $\gamma^{\left(i\right)}=\gamma$ for each cycle. The above equation can be further simplified as, noticing $\eta_{C}\left(t\right)=1-T_{L}^{\left(0\right)}/T_{H}\left(t\right)$ and $dQ_{H}=-C_{H}dT_{H}$,

(32) $$\eta_{U}^{\mathrm{FT}}=\frac{\int_{T_{H}^{\left(0\right)}}^{T_{L}^{\left(0\right)}}\frac{T_{H}\left(t\right)-T_{L}^{\left(0\right)}}{\left(2-\gamma \right)T_{H}\left(t\right)+\gamma T_{L}^{\left(0\right)}}dT_{H}}{\int_{T_{H}^{\left(0\right)}}^{T_{L}^{\left(0\right)}}dT_{H}},$$

After finishing the integral, the upper bound for efficiency is finally found as, in terms of the initial Carnot efficiency $\eta_{C}$,

(33) $$\eta^{\mathrm{FT}}\left(\xi \to \infty \right)=\frac{1}{2-\gamma }\left[1-\frac{2(1-\eta_{C})}{\left(2-\gamma \right)\eta_{C}}\ln\frac{2-\gamma \eta_{C}}{2(1-\eta_{C})}\right],$$

which is a monotonically increasing function of γ, since

(34) $$\frac{\partial \eta^{\mathrm{FT}}\left(\xi \to \infty \right)}{\partial \gamma }=\frac{1}{{\left(2-\gamma \right)}^{2}}\left[1+\frac{4(1-\eta_{C})}{\left(2-\gamma \right)\left(2-\gamma \eta_{C}\right)}\right]>0$$

**Figure 5:**
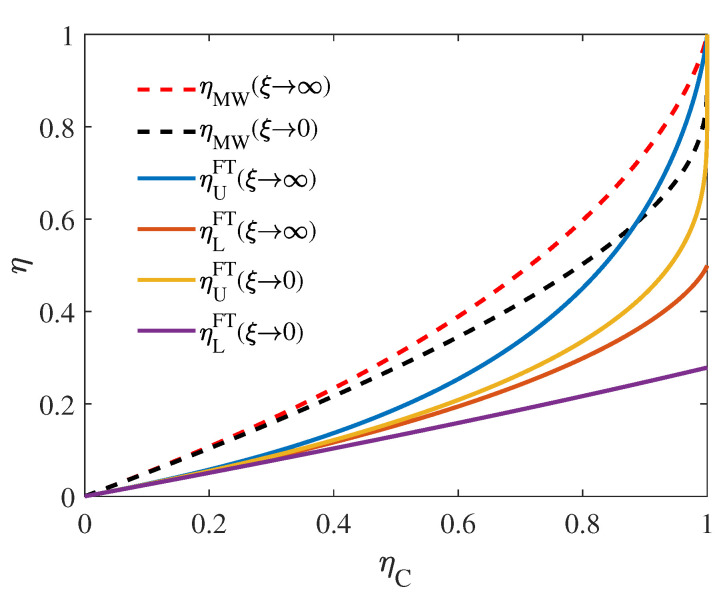
Upper ($\eta_{U}^{\mathrm{FT}}$) and lower ($\eta_{L}^{\mathrm{FT}}$) bounds for efficiency of the heat engine in finite-time operation as the function of $\eta_{C}$ in different limit of ξ. $\eta_{U}^{\mathrm{FT}}$ and $\eta_{L}^{\mathrm{FT}}$ respectively correspond to γ→1 and γ→0. Here ξ and γ respectively characterize the asymmetry in size and in dissipation of the two heat sources. As the comparison, the red(black) dashed line represent the upper (lower) bound of EMP in the reversible limit given by Equation (13). Other are plotted with Equations (38)–(41).

Thus, $\eta^{\mathrm{FT}}\left(\xi \to \infty \right)$ is bounded by the following inequality

(35) $$\eta_{L}^{\mathrm{FT}}\left(\xi \to \infty \right)\leq \eta^{\mathrm{FT}}\left(\xi \to \infty \right)\leq \eta_{U}^{\mathrm{FT}}\left(\xi \to \infty \right),$$

where the upper bound

(36) $$\eta_{U}^{\mathrm{FT}}\left(\xi \to \infty \right)=1-\frac{2(1-\eta_{C})}{\eta_{C}}\ln\frac{2-\eta_{C}}{2(1-\eta_{C})}.$$

is obtained by taking γ=1, while

(37) $$\eta_{L}^{\mathrm{FT}}\left(\xi \to \infty \right)=\frac{1}{2}\left[1+\left(\eta_{C}^{-1}-1\right)\ln\left(1-\eta_{C}\right)\right],$$

is the lower bound of $\eta^{\mathrm{FT}}\left(\xi \to \infty \right)$, which is achieved by taking γ=0, and is exactly half of $\eta_{\mathrm{MW}}\left(\xi \to \infty \right)$ in Equation (13) in the reversible case. Similarly, in the limit of ξ→0, another two bounds for $\eta^{\mathrm{FT}}$ are obtained and the corresponding derivation are given in Appendix B. Here we make a brief summary of the bounds for $\eta^{\mathrm{FT}}$ obtained in different limit as follows

(38) $$\eta_{U}^{\mathrm{FT}}\left(\xi \to \infty \right)=1+\frac{2(1-\eta_{C})}{\eta_{C}}\ln\frac{2(1-\eta_{C})}{2-\eta_{C}},$$

(39) $$\eta_{L}^{\mathrm{FT}}\left(\xi \to \infty \right)=\frac{\eta_{\mathrm{MW}}\left(\xi \to \infty \right)}{2},$$

(40) $$\eta_{U}^{\mathrm{FT}}\left(\xi \to 0\right)=\frac{\eta_{\mathrm{MW}}\left(\xi \to 0\right)}{2-\eta_{\mathrm{MW}}\left(\xi \to 0\right)},$$

(41) $$\eta_{L}^{\mathrm{FT}}\left(\xi \to 0\right)=1+\frac{\eta_{C}}{2}\ln^{-1}\left(1-\frac{\eta_{C}}{2}\right),$$

where $\eta_{\mathrm{MP}}\left(\xi \to \infty \right)$ and $\eta_{\mathrm{MP}}\left(\xi \to 0\right)$ are the corresponding efficiency in the reversible limit as given by Equation (13). Interestingly, the dependence of η on $\eta_{\mathrm{MW}}$ in the limit of $\left(\xi \to \infty ,\gamma \to 0\right)$ and $\left(\xi \to 0,\gamma \to 1\right)$ follows the same form as its corresponding counterpart in the infinite heat source case: the lower bound of the efficiency at maximum power follows $\eta_{C}/2$, while the upper bound of the efficiency at maximum power follows $\eta_{C}/\left(2-\eta_{C}\right)$. These efficiency in different limit of ξ and γ are illustrated in Figure 5 with the solid lines, where the dashed lines are the upper and lower bounds of EMW, i.e., $\eta_{\mathrm{MW}}\left(\xi \to \infty \right)$ and $\eta_{\mathrm{MW}}\left(\xi \to 0\right)$. It is easily to check that these efficiency follow the universality as

(42) $$\eta^{\mathrm{FT}}=\frac{\eta_{C}}{4}+O\left(\eta_{C}^{2}\right).$$

Note that $\eta_{\mathrm{MW}}$ can be regarded as the limit efficiency of $\eta^{\mathrm{FT}}$ at the quasi-static limit with no irreversibility. In finite-time thermodynamics, for a heat engine working between two constant temperature heat sources (heat capacity of the sources approach infinite), the universality of the efficiency at maximum power $\eta_{\mathrm{MP}}$ is generally written as a function of $\eta_{C}$(quasi-static limit), as

(43) $$\eta_{\mathrm{MP}}=\frac{\eta_{C}}{2}+O\left(\eta_{C}^{2}\right)$$

Similarly, comparing Equation (42) with Equation (24), we can also write the universality of $\eta^{\mathrm{FT}}$ in term of $\eta_{\mathrm{MW}}$ as

(44) $$\eta =\frac{\eta_{\mathrm{MW}}}{2}+O\left(\eta_{\mathrm{MW}}^{2}\right),$$

which means that, up to the first order of $\eta_{C}$, the efficiency when the power of each cycle is maximized is just half of the EMW. Such universality is also found for the efficiency at maximum time-average power with linear irreversible heat engine under the tight coupling condition [52,53].

## 4. Black Holes Served as Heat Sources

In the previous section, we have discussed the situation that heat engine operates to stop between two finite-size heat sources when the high-temperature and low-temperature source reach the same temperature. This actually based on the assumption that the heat capacity of the heat sources are positive as we mentioned before, such that the temperature of the high-temperature heat source is lowered and the temperature of the low-temperature heat source is increased as the engine’s working. Although most physical systems have positive heat capacity, there are indeed some systems with negative heat capacity, i.e., C=∂U/∂T<0, such as black holes [68,69]. All the thermodynamic properties of a black hole only rely on its mass *M*, angular momentum *J*, and charge *Q*, known as the three hairs of black hole [69]. For simplicity, we consider the Schwarzschild black hole, which only has one hair, the mass. The internal energy and temperature of a Schwarzschild black hole B with mass *M* are respectively U=M and $T=1/\left(8\pi M\right)$. Here and after, we use the natural unit system. Therefore, the heat capacity of B is $C=\partial U/\partial T=-8\pi M^{2}$. Obviously, such heat capacity is negative and increases quadratically with the black hole’s mass.

Now we consider two Schwarzschild black holes of mass $M_{H}$ and $M_{L}$ served as high and low temperature heat source, respectively. Note that $T_{H}=1/\left(8\pi M_{H}\right)$ should be higher than $T_{L}=1/\left(8\pi M_{L}\right)$, thus the high temperature black hole has smaller mass than the low temperature one, namely, $M_{H}<M_{L}$. The working substance reciprocates between the two black holes and exchanges heat as well as output work, and we ignore the influence of gravity on the cycle process in the following discussion. After the heat engine absorbs heat from the high temperature black hole, the mass of the high temperature black hole decreases, i.e., $M_{H}↓$ and then its temperature rises, namely $T_{H}↑$; and when the heat is released to the low-temperature black hole, the mass $M_{C}↑$ and the temperature $T_{L}↓$ consequently. This is exactly the opposite of what we discussed for the positive heat capacity bath. Thus, the condition that the heat engine stops working is no longer the temperature convergence of the two heat sources ($T_{L}\left(t_{f}\right)=T_{H}\left(t_{f}\right)\equiv T_{E}$), but the high temperature heat source, i.e., the smaller black hole, is exhausted, namely, $M_{H}\left(t_{f}\right)=0$. As a result, the efficiency of the heat engine work between these two black holes follows

(45) $$\eta =1-\frac{M_{L}\left(t_{f}\right)-M_{L}}{M_{H}}.$$

Here we have assumed that, apart from exchanging heat, the black holes and heat engine have no other energy exchange channels. We still consider the reversible cycle for convenient, and the the reversible condition of Equation (10) now becomes

(46) $$\int_{M_{H}^{\left(0\right)}}^{0}\frac{dM_{H}}{1/\left(8\pi M_{H}\right)}+\int_{M_{L}^{\left(0\right)}}^{M_{L}\left(t_{f}\right)}\frac{dM_{L}}{1/\left(8\pi M_{L}\right)}=0,$$

where $M_{H}^{\left(0\right)}$ and $M_{L}^{\left(0\right)}$ are the initial mass of these two black holes. Then we obtain

(47) $${\left(M_{H}^{\left(0\right)}\right)}^{2}+{\left(M_{L}^{\left(0\right)}\right)}^{2}=M_{L}^{2}\left(t_{f}\right).$$

**Figure 6:**
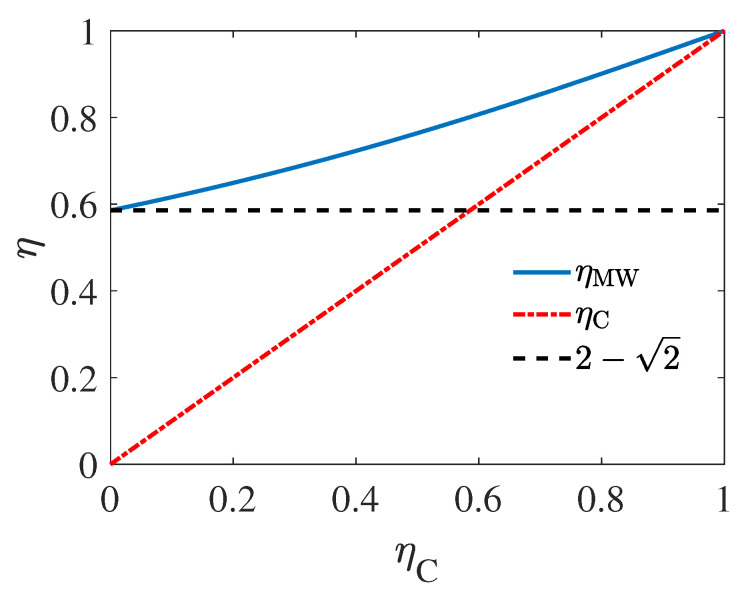
Efficiency at maximum work $\eta_{\mathrm{MW}}$ of a heat engine working between two Schwarzschild black holes.

This result has been obtained in a recent work [70], where the black hole and corresponding radiation are considered as the heat reservoir. In addition, Equation (47)can also be derived with the conservation of black hole area entropy with no information loss [70,71,72,73,74] as

(48) $$\sum_{\alpha =H,C}S_{\mathrm{BH}}\left(M_{\alpha }^{\left(0\right)}\right)=\sum_{\alpha =H,C}S_{\mathrm{BH}}\left[M_{\alpha }\left(t_{f}\right)\right]$$

with $S_{\mathrm{BH}}\left(M\right)=4\pi M^{2}$ being the Beckenstein-Hawking Entropy. Combining Equations (45) and (47), the EMW of this case reads

(49) $$\eta_{\mathrm{MW}}=1-\frac{\sqrt{{\left(1-\eta_{C}\right)}^{2}+1}-1}{1-\eta_{C}}$$

where

(50) $$\eta_{C}=1-\frac{T_{L}^{\left(0\right)}}{T_{H}^{\left(0\right)}}=1-\frac{M_{H}^{\left(0\right)}}{M_{L}^{\left(0\right)}}.$$

is the Carnot efficiency defined by the initial mass of the two black holes. Note that

(51) $$\begin{array}{cc} \eta_{\mathrm{MW}} & =\frac{2-\eta_{C}-\sqrt{{\left(1-\eta_{C}\right)}^{2}+1}}{1-\eta_{C}}\\ \; & =\eta_{C}+\frac{{\left(1-\eta_{C}\right)}^{2}+1-\sqrt{{\left(1-\eta_{C}\right)}^{2}+1}}{1-\eta_{C}}\\ \; & \geq \eta_{C} \end{array}$$

which implies that the $\eta_{\mathrm{MW}}$ of the heat engine can surpass the initial Carnot efficiency due to the unusual nature of negative heat capacity of black hole. $\eta_{\mathrm{MW}}$ as the function of $\eta_{C}$ is plotted in Figure 6, where $\eta_{\mathrm{MW}}$ can be found to be obviously higher than $\eta_{C}$. We should emphasize here that this result does not violate the second law of thermodynamics, since the temperature of the two black holes are not constant, but has an increasing temperature difference as the heat engine works. Such that, the efficiency of the cycle, which is a monotonically increasing function of the temperature difference of the sources, will also increase as time goes by. Moreover, compared with the result relates to positive heat capacity sources in Equations (12) and (17), the EMW of Equation (49) only rely on the initial Carnot efficiency, i.e., $\eta_{C}$, without relying on other parameters. Since $\eta_{C}$ only depends on the initial mass of the two black holes, as shown by Equation (50), we can regard this result as the embodiment of the No-hair theorem [69], which states that the features of black holes are uniquely determined by only a few parameters of the black holes, namely, mass, charge, and angular momentum, in efficiency for heat engines operating between black holes.

When the initial mass of the two black holes are the same, i.e., $M_{H}^{\left(0\right)}=M_{L}^{\left(0\right)}$ and $\eta_{C}=0$, the heat engine can be driven with some energy fluctuation near the horizon of the black hole due to the Hawing radiation process [75,76]: due to energy fluctuations, black holes of the same mass may have a slight temperature difference, and the heat engine is then driven. As we mentioned before, the temperature difference between black holes will be amplified as the heat engine works. In such circumstance, one has

(52) $$\lim_{\eta_{C}\to 0}\eta_{\mathrm{MW}}=2-\sqrt{2}.$$

The above limit is represented by the black dashed line in Figure 6. We note that there are studies that have linked black holes to heat engines [77,78,79]. However, to our best knowledge, these works mainly consider the black holes as the working substance rather than heat sources, and do not consider the finite-size effect.

Besides the black hole, the negative heat capacity also been observed in some Cluster of atom system with phase transition [80,81,82]. The discussion in this section can be extends to these systems, we hope to use these novel materials to achieve high energy conversion efficiency in the near future.

## 5. Conclusions and Discussion

In summary, we studied the efficiency of a heat engine working between two finite-size heat sources in both quasi-static and finite-time cases. The effect of the heat capacity of the finite-size heat source on the engine’s efficiency was clarified. When the engine operates in quasi-static cycles, with the assumption that the source’s capacity follows the Debye’s law, we obtained the corresponding efficiency at maximum work (EMW) in the high and low temperature regime, as given by Equation (12) and Equation (17), respectively. We also found some bounds for such EMW in different circumstances (Equation (13) of high temperature regime and Equations (19) and (20) for low temperature). We proved that, in the low temperature case, with the limit of $\eta_{C}\to 1$, the lower bound of $\eta_{\mathrm{EMW}}$ only determined by the heat source’s dimension, i.e., $\lim_{\eta_{C}\to 1}\eta_{\mathrm{MW}}\left(\xi \to 0\right)={\left(n+1\right)}^{-1}$. Particularly, for the 1-dimensional sources, the upper and lower bound are obtained as $\eta_{U}=\eta_{C}{\left(2-\eta_{C}\right)}^{-1}$ and $\eta_{L}=\eta_{C}/2$, respectively. We noted that some results of Section 2, for example Equation (22), have been reported in Ref. [55]. However, Ref. [55] uses the energy-entropy relation, while our consideration it based on the perspective of heat capacity. Moreover, to our best knowledge, the universality for EMW, namely, $\eta_{C}/4+O\left(\eta_{C}^{2}\right)$ was first given in this paper.

For the heat engine working within finite time, we modeled the engine as a low-dissipation Carnot-like engine and studied the efficiency with the output power of each cycle is maximized. A series of bounds for the efficiency are obtained in Equations (38)–(41) and plotted in Figure 5, where the overall upper bound is achieved with ξ→∞,γ→1, namely, the heat capacity of cold source is much lager than that of the hot one while the dissipation between the cold source and the heat engine approaches vanish. An universality was found for all these bounds obtained with finite-size sources and finite-time as $\eta =\eta_{C}/4+O\left(\eta_{C}^{2}\right)$, where the coefficient of $\eta_{C}$’s first order is half of that of the EMP and EMW. Unlike the optimization in Refs. [52,53], where the efficiency at maximum time-average power of the whole process is studied, in this work, we focus on how efficient the engine can be when the power of each cycle is maximized.

Although we have discussed the effect of asymmetry in the size of the hot and cold sources on the efficiency, the results are obtained for the heat capacity function rely on temperature of the sources share the same form. Considering that the heat capacity of the high and low temperature sources have different temperature dependence, such as cold source’s heat capacity varies with temperature following the power law while the capacity of the hot source heat capacity remains constant, etc., is a potential direction for the optimization of the heat engine’s performance. In addition, the effect of phase transition (PT) is also worth exploring in future study, as the heat capacity of sources with PT may have completely different characteristics in different phases due to temperature changes. Note that, recently, PT of the working substance has been found to be beneficial to the heat engines’ performance [22,83,84].

In the last part of this paper, we studied an unusual case where the sources have negative heat capacity. Using black holes as a demonstration, we obtain the EMW for a heat engine working between two Schwarzschild black holes as shown in Equation (6). The EMW is found always higher than the initial Carnot efficiency defined by the initial mass of the two black holes. However, we emphasize that this does not violate the second law of thermodynamics, since the temperature of the two black hole are not constant but has an increasing temperature difference as the heat engine operates due to the negative heat capacity of the sources. In addition, even when the initial mass of the two black holes are the same, the heat engine can be driven with some energy fluctuation between the working substance and one of the black hole from the Hawing radiation process. In this situation, the EMW is proved to be $\eta_{\mathrm{MW}}=2-\sqrt{2}$. We note that the output-work in such situation is also studied in Ref. [70]. However, our work mainly focus on the EMW of the thermodynamic process, and emphasizes that for negative heat capacity systems such as black holes, EMW can exceed the Carnot efficiency determined by the initial heat source temperature, which have not been reported before.

In this paper, we only studied the quasi-static heat engines working between the Schwarzschild black holes, and the black holes only exchange heat with the working substance without external energy transfer. Considering the finite-time effect, the non-negligible energy loss due to the Hawking radiation, the cases with other types of black holes, and the gravitational effect on the efficiency of the heat engine will be a series of interesting and challenging tasks, which will be investigated in our further studies. Moreover, since there have been some experimental reports on negative heat capacity materials [80,81,82], it is feasible to take use of these materials as heat sources providing energy for the heat engines to test our predictions.

## Figures and Tables

**Table 1:** Bound for efficiency in different case. Here *t* is the operation time of the heat engine and *C* is the heat capacity of the heat source. In the case engine working in quasi-static cycle between infinite heat bath, i.e., t→∞, C→∞, the maximum achievable efficiency, as stated by Carnot, is the Carnot efficiency $\eta_{C}=1-T_{L}/T_{H}$. For the engine operates in finite time, i.e., C→∞, t↛∞, Esposito et al. [41] give the bounds for the efficiency at maximum power (EMP), $\eta_{\mathrm{MP}}$, with low-dissipation Carnot-like engine. The bounds for efficiency at maximum work (EMW) (t→∞, C↛∞), $\eta_{\mathrm{MW}}$, and efficiency at maximum power for each cycle (t↛∞, C↛∞), $\eta^{\mathrm{FT}}$, are obtained in this paper. The detailed derivations are illustrated in Section 2.1 and Section 3, respectively. The bounds in the latter two cases for $\eta_{\mathrm{MW}}$ and $\eta^{\mathrm{FT}}$ in this table are limited to the heat source having a positive and constant heat capacity. The bounds correspond to heat capacity change with temperature are discussed in Section 2.2 while the negative heat capacity case are discussed in Section 4.

	t→∞	t↛∞
C→∞	$\eta_{\max}=\eta_{C}$	$\frac{\eta_{C}}{2}\leq \eta_{\mathrm{MP}}\leq \frac{\eta_{C}}{2-\eta_{C}}$
C↛∞	$1+\frac{\left(1-\eta_{C}\right)\ln\left(1-\eta_{C}\right)}{\eta_{C}}\leq \eta_{\mathrm{MW}}\leq 1+\frac{\eta_{C}}{\ln\left(1-\eta_{C}\right)}$	$1+\frac{\eta_{C}/2}{\ln\left(1-\eta_{C}/2\right)}\leq \eta^{\mathrm{FT}}\leq 1+\frac{1-\eta_{C}}{\eta_{C}/2}\ln\frac{1-\eta_{C}}{1-\eta_{C}/2}$

## Appendix A. Optimization of Low-Dissipation Carnot-Like Engine

In this Appendix, we briefly show the optimization of the low-dissipation Carnot-like engine introduced in Section 3, we follow the same optimization method in Ref. [41]. For such engine working in the *i*-th Carnot-like cycle, the heat transfer in the two finite-time isothermal processes read

(A1) $$Q_{H}^{\left(i\right)}=T_{H}^{\left(i\right)}S_{\mathrm{re}}^{\left(i\right)}-\frac{\Gamma_{H}^{\left(i\right)}}{\tau_{H}^{\left(i\right)}},$$

and

(A2) $$Q_{L}^{\left(i\right)}=T_{L}^{\left(i\right)}S_{\mathrm{re}}^{\left(i\right)}+\frac{\Gamma_{L}^{\left(i\right)}}{\tau_{L}^{\left(i\right)}},$$

where, $T_{H}^{\left(i\right)}$($T_{L}^{\left(i\right)}$) is the temperature of the high (low) temperature heat source, $S_{\mathrm{re}}^{\left(i\right)}$ is reversible entropy change, $\Gamma_{\alpha }^{\left(i\right)}$ depends on the dissipative nature of the working substance when contacting with the heat source and $\tau_{\alpha }^{\left(i\right)}$ is the corresponding operation time. Thus, the efficiency and power of the engine for the *i*-th cycle follows

(A3) $$\begin{array}{cc} \eta^{\left(i\right)} & =\frac{Q_{H}^{\left(i\right)}-Q_{L}^{\left(i\right)}}{Q_{H}^{\left(i\right)}}=\frac{\left(T_{H}^{\left(i\right)}-T_{L}^{\left(i\right)}\right)S_{\mathrm{re}}^{\left(i\right)}-\frac{\Gamma_{H}^{\left(i\right)}}{\tau_{H}^{\left(i\right)}}-\frac{\Gamma_{L}^{\left(i\right)}}{\tau_{L}^{\left(i\right)}}}{T_{H}^{\left(i\right)}S_{\mathrm{re}}^{\left(i\right)}-\frac{\Gamma_{H}^{\left(i\right)}}{\tau_{H}^{\left(i\right)}}}, \end{array}$$

and

(A4) $$\begin{array}{cc} P^{\left(i\right)} & =\frac{\Delta Q_{H}^{\left(i\right)}-\Delta Q_{L}^{\left(i\right)}}{\tau_{H}^{\left(i\right)}+\tau_{L}^{\left(i\right)}}=\frac{\left(T_{H}^{\left(i\right)}-T_{L}^{\left(i\right)}\right)\Delta S_{\mathrm{re}}^{\left(i\right)}-\frac{\Gamma_{H}^{\left(i\right)}}{\tau_{H}^{\left(i\right)}}-\frac{\Gamma_{L}^{\left(i\right)}}{\tau_{L}^{\left(i\right)}}}{\tau_{H}^{\left(i\right)}+\tau_{L}^{\left(i\right)}} \end{array}$$

Here the operation time in the two adiabatic processes are ignored [41]. The maximum power of each cycle is obtained by setting the derivatives of $P^{\left(i\right)}=P^{\left(i\right)}\left(\tau_{H}^{\left(i\right)},\tau_{L}^{\left(i\right)}\right)$ with respect to $\tau_{H}^{\left(i\right)}$ and $\tau_{L}^{\left(i\right)}$ equal to zero. Thus we find the corresponding times for the engine working at maximum power as

(A5) $$\tau_{H}^{\left(i\right)}=2\frac{\Gamma_{H}^{\left(i\right)}}{\left(T_{H}^{\left(i\right)}-T_{L}^{\left(i\right)}\right)S_{\mathrm{re}}^{\left(i\right)}}\left(1+\sqrt{\frac{\Gamma_{L}^{\left(i\right)}}{\Gamma_{H}^{\left(i\right)}}}\right)$$

and

(A6) $$\tau_{L}^{\left(i\right)}=\tau_{H}^{\left(i\right)}\sqrt{\frac{\Gamma_{L}^{\left(i\right)}}{\Gamma_{H}^{\left(i\right)}}}$$

Substituting Equations (A5) and (A6) into Equation (A3), the efficiency at maximum power is obtained as

(A7) $$\eta_{\mathrm{MP}}^{\left(i\right)}=\frac{\eta_{C}^{\left(i\right)}}{2-\gamma^{\left(i\right)}\eta_{C}^{\left(i\right)}},$$

where

(A8) $$\gamma^{\left(i\right)}={\left(1+\sqrt{\Gamma_{L}^{\left(i\right)}/\Gamma_{H}^{\left(i\right)}}\right)}^{-1}$$

and $\eta_{C}^{\left(i\right)}=1-T_{L}^{\left(i\right)}/T_{H}^{\left(i\right)}$ is the Carnot efficiency determined by the temperature of the sources in the *i*-th cycle

## Appendix B. Bounds for Efficiency in Finite Time in the Limit of ξ→0

In this Appendix, we derive the upper and lower bounds for efficiency of heat engine working between finite-size source within finite time in the limit of ξ→0. In this case, the size of the cold source is much smaller than that of the hot one, thus $T_{H}^{\left(i\right)}=T_{H}^{\left(0\right)}$ and $T_{L}^{\left(N\right)}=T_{H}^{\left(0\right)}$ at $t=t_{f}$. In the limit of γ→1, the efficiency of Equation (30) becomes

(A9) $$\eta =\frac{\sum_{i=1}^{N}\frac{\eta_{C}^{\left(i\right)}}{2-\eta_{C}^{\left(i\right)}}Q_{H}^{\left(i\right)}}{\sum_{i=1}^{N}Q_{H}^{\left(i\right)}}.$$

Note that in this limit, Equations (A5) and (A6) respectively reduce to $\tau_{H}^{\left(i\right)}=2\Gamma_{H}^{\left(i\right)}/\left(T_{H}^{\left(i\right)}\eta_{C}^{\left(i\right)}S_{\mathrm{re}}^{\left(i\right)}\right)$ and $\tau_{L}^{\left(i\right)}=0$, substituting which into Equation (A1) and Equation (A2), we find the relation between heat transfer and reversible entropy change as

(A10) $$\begin{array}{cc} \Delta Q_{H}^{\left(i\right)} & =T_{H}^{\left(i\right)}S_{\mathrm{re}}^{\left(i\right)}\left(1-\frac{\eta_{C}^{\left(i\right)}}{2}\right). \end{array}$$

(A11) $$\begin{array}{cc} \; & =T_{H}^{\left(0\right)}S_{\mathrm{re}}^{\left(i\right)}\left(1-\frac{\eta_{C}^{\left(i\right)}}{2}\right). \end{array}$$

and

(A12) $$\Delta Q_{L}^{\left(i\right)}=T_{L}^{\left(i\right)}S_{\mathrm{re}}^{\left(i\right)}.$$

Using Equations (A11) and (A12), and replace the sum by integral with N≫1, Equation (A9) is simplified as

(A13) $$\begin{array}{cc} \eta & =\frac{\int_{0}^{t_{f}}\frac{\eta_{C}\left(t\right)}{2-\eta_{C}\left(t\right)}\left[1-\frac{\eta_{C}\left(t\right)}{2}\right]T_{H}^{\left(0\right)}{\dot{S}}_{\mathrm{re}}dt}{\int_{0}^{t_{f}}\left[1-\frac{\eta_{C}\left(t\right)}{2}\right]T_{H}^{\left(0\right)}{\dot{S}}_{\mathrm{re}}dt} \end{array}$$

(A14) $$\begin{array}{cc} \; & =\frac{\int_{0}^{t_{f}}\eta_{C}\left(t\right){\dot{S}}_{\mathrm{re}}dt}{\int_{0}^{t_{f}}\left[2-\eta_{C}\left(t\right)\right]{\dot{S}}_{\mathrm{re}}dt} \end{array}$$

(A15) $$\begin{array}{cc} \; & =\frac{\int_{T_{L}^{\left(0\right)}}^{T_{H}^{\left(0\right)}}\eta_{C}\left(t\right)\left(\frac{C_{L}dT_{L}}{T_{L}}\right)}{\int_{T_{L}^{\left(0\right)}}^{T_{H}^{\left(0\right)}}\left[2-\eta_{C}\left(t\right)\right]\left(\frac{C_{L}dT_{L}}{T_{L}}\right)} \end{array}$$

(A16) $$\begin{array}{cc} \; & =\frac{\int_{T_{L}^{\left(0\right)}}^{T_{H}^{\left(0\right)}}\left(\frac{T_{H}^{\left(0\right)}}{T_{L}}-1\right)dT_{L}}{\int_{T_{L}^{\left(0\right)}}^{T_{H}^{\left(0\right)}}\left(\frac{T_{H}^{\left(0\right)}}{T_{L}}+1\right)dT_{L}} \end{array}$$

By straightforward calculation, we have

(A17) $$\eta_{U}^{\mathrm{FT}}\left(\xi \to 0\right)=1-\frac{2\eta_{C}}{\eta_{C}-\ln\left(1-\eta_{C}\right)},$$

which can be re-expressed with $\eta_{\mathrm{MW}}\left(\xi \to 0\right)$ of Equation (13) as

(A18) $$\eta_{U}^{\mathrm{FT}}\left(\xi \to 0\right)=\frac{\eta_{\mathrm{EMW}}\left(\xi \to 0\right)}{2-\eta_{\mathrm{EMW}}\left(\xi \to 0\right)}$$

This is the upper bound for ξ→0 we illustrated in Equation (40). On the other hand, for γ→0, Equation (30) becomes,

(A19) $$\begin{array}{cc} \eta & =\frac{\int_{0}^{t_{f}}\frac{\eta_{C}\left(t\right)}{2}T_{H}^{\left(0\right)}{\dot{S}}_{\mathrm{re}}dt}{\int_{0}^{t_{f}}T_{H}^{\left(0\right)}{\dot{S}}_{\mathrm{re}}dt} \end{array}$$

(A20) $$\begin{array}{cc} \; & =\frac{\int_{T_{L}^{\left(0\right)}}^{T_{H}^{\left(0\right)}}\frac{\eta_{C}\left(t\right)}{2}T_{H}^{\left(0\right)}\left[\frac{2-2\eta_{C}\left(t\right)}{2-\eta_{C}\left(t\right)}\right]\left(\frac{C_{L}dT_{L}}{T_{L}}\right)}{\int_{T_{L}^{\left(0\right)}}^{T_{H}^{\left(0\right)}}T_{H}^{\left(0\right)}\left[\frac{2-2\eta_{C}\left(t\right)}{2-\eta_{C}\left(t\right)}\right]\left(\frac{C_{L}dT_{L}}{T_{L}}\right)} \end{array}$$

(A21) $$\begin{array}{cc} \; & =\frac{\int_{T_{L}^{\left(0\right)}}^{T_{H}^{\left(0\right)}}\frac{T_{H}^{\left(0\right)}-T_{L}}{T_{H}^{\left(0\right)}+T_{L}}dT_{L}}{2\int_{T_{L}^{\left(0\right)}}^{T_{H}^{\left(0\right)}}\frac{1}{T_{H}^{\left(0\right)}+T_{L}}dT_{L}}, \end{array}$$

where we have used

(A22) $$\Delta Q_{H}^{\left(i\right)}=T_{H}^{\left(i\right)}\Delta S_{\mathrm{re}}^{\left(i\right)}=T_{H}^{\left(0\right)}\Delta S_{\mathrm{re}}^{\left(i\right)}$$

and

(A23) $$\Delta Q_{L}^{\left(i\right)}=T_{L}^{\left(i\right)}\Delta S_{\mathrm{re}}^{\left(i\right)}\left(\frac{2-\eta_{C}^{\left(i\right)}}{2-2\eta_{C}^{\left(i\right)}}\right),$$

in the limit of γ→0. Finish the integral of Equation (A21), we obtain

(A24) $$\eta_{L}^{\mathrm{FT}}\left(\xi \to 0\right)=1+\frac{\eta_{C}}{2}\ln^{-1}\left(1-\frac{\eta_{C}}{2}\right),$$

which is the lower bound $\eta^{\mathrm{FT}}$ of in the limit of ξ→0 as illustrated in Equation (40).
